# Breeding in the Economically Important Brown Alga *Undaria pinnatifida*: A Concise Review and Future Prospects

**DOI:** 10.3389/fgene.2021.801937

**Published:** 2021-12-01

**Authors:** Tifeng Shan, Shaojun Pang

**Affiliations:** ^1^ CAS Key Laboratory of Experimental Marine Biology, Institute of Oceanology, Chinese Academy of Sciences, Qingdao, China; ^2^ Center for Ocean Mega-Science, Chinese Academy of Sciences, Qingdao, China; ^3^ Laboratory for Marine Biology and Biotechnology, Qingdao National Laboratory for Marine Science and Technology, Qingdao, China

**Keywords:** kelp, Laminariales, seaweed, brown alga, cultivar breeding, wakame, genetic improvement, life history

## Abstract

*Undaria pinnatifida* is the commercially second most important brown alga in the world. Its global annual yield has been more than two million tonnes since 2012. It is extensively cultivated in East Asia, mainly consumed as food but also used as feed for aquacultural animals and raw materials for extraction of chemicals applicable in pharmaceutics and cosmetics. Cultivar breeding, which is conducted on the basis of characteristics of the life history, plays a pivotal role in seaweed farming industry. The common basic life history shared by kelps determines that their cultivar breeding strategies are similar. Cultivar breeding and cultivation methods of *U. pinnatifida* have usually been learned or directly transferred from those of *Saccharina japonica*. However, recent studies have revealed certain peculiarity in the life history of *U. pinnatifida*. In this article, we review the studies relevant to cultivar breeding in this alga, including the peculiar component of the life history, and the genetics, transcriptomics and genomics tools available, as well as the main cultivar breeding methods. Then we discuss the prospects of cultivar breeding based on our understanding of this kelp and what we can learn from the model brown alga and land crops.

## Introduction

The brown alga *Undaria pinnatifida* (Harvey) Suringar is an economically important kelp species native to the Northwest Pacific and has been extensively farmed as human food in East Asia for more than half a century (Yamanaka and Akiyama, 1993). It also can be used as feed for aquacultural animals and raw materials for extraction of chemicals applicable in pharmaceutics and cosmetics (Hwang et al., 2012; Taboada et al., 2012; Yang et al., 2013; Arijón et al., 2021). Its global annual yield has been more than two million tonnes since 2012, second to *Saccharina japonica* in commercial brown algae (Cai et al., 2021). In addition to East Asia, there is also small-scale cultivation of this alga in Europe, mainly in France and Spain (Grulois et al., 2011; Peteiro and Freire, 2011, 2012).

Domestication and commercial cultivation of kelps has generally been started since 1950s (Tseng et al., 1955). Cultivar breeding has been playing an important role in accelerating the development of kelp cultivation industry (Hwang et al., 2019; Wang et al., 2020; Hu et al., 2021). *U. pinnatifida* and *S. japonica* share the same basic life history typical of kelp species, which determines that their cultivar breeding strategies are similar and may be referenced to each other. A large proportion of the *Undaria* products produced in China and Korea are exported to Japan and thus the purposes of cultivar breeding are heavily dependent on requirements of the Japanese market (Hwang et al., 2019). Compared to *S. japonica*, the agronomical traits of *U. pinnatifida* are more complex, e.g. with pinnate fronds and the specific reproductive tissue named sporophyll. Furthermore, quality of the fronds is more demanding, with smooth, thick and brightly yellowish-brown ones being preferred in commerce. Hence, cultivar breeding aims are more diverse, and it is difficult to breed an elite cultivar encompassing all the desirable traits.

In spite of the same basic life history shared between *U. pinnatifida* and other kelps, recent studies have revealed certain peculiarity in the life history of *U. pinnatifida* (Li et al., 2014). The new technology, such as the next-generation sequencing, has been increasingly applied in studies on genetics, transcriptomics, and genomics of this alga, providing valuable information for cultivar breeding. The purpose of this article is to review the research relevant to cultivar breeding of *U. pinnatifida* with special highlight on its peculiarity and discuss the prospects.

## Life History

*U. pinnatifida* has a basic haplodiplontic life history in which diploid macroscopic sporophytes (2n) alternate with haploid microscopic gametophytes (n). Mature sporophytes generate haploid spores through meiosis. The meiospores grow to male and female gametophytes, which are usually dioicous and give rise to sperm and eggs through mitosis during gametogenesis, respectively. The discharged eggs are attached to the somatic cells, while the motile sperm swarm to the eggs under the induction of pheromones released by the female gametophytes (Maier and Müller, 1986). After the eggs are fertilized by the sperm, zygotes are formed and the sporophyte phase is started again.

Some unfertilized eggs are able to develop to sporophytes through parthenogenesis, a phenomenon that occurs supposedly mainly depending on genotype of the female gametophyte (Fang et al., 1979; Nakahara, 1984; Shan et al., 2013). Most of the parthenosporophytes are abnormal (e.g., twisted, or asymmetrical or not intact) due to mixed ploidy (chimera). However, a small proportion of them is normal and diploid, and these sporophytes can become mature and release spores, all of which grow to female gametophytes (Fang et al., 1979; Shan et al., 2013). Spontaneous chromosome doubling is speculated to happen in diploid parthenosporophytes (Fang et al., 1984). In addition, apogamy, which is referred to the emergence of sporophytes directly from the somatic cells of gametophytes, i.e., without going through gametogenesis, was also described in some male gametophytes (Fang and Dai, 1980). In *S. japonica*, the sporophytes derived from apogamy were mostly abnormal and haploid, and they could not form sporangia (Dai et al., 1997). However, there has been no report on the fate of the sporophytes derived from apogamy in *U. pinnatifida*.

Recently, an unusual monoicous phenomenon has been found in some zoospore-derived male gametophyte clonal lines (Li et al., 2014). They show typical male morphology during vegetative growth; however, when cultured under conditions favoring gametogenesis both antheridia and oogonia will be formed, discharging sperm and eggs, respectively. The monoicous gametophytes lack the female-linked markers (Li et al., 2017). They are determined to be haploid by flow cytometry analysis, different from the diploid gametophyte derived from tissue culture of the sporophyte thalli (Zhang et al., 2007; Shan et al., 2020b). Microsatellite analysis confirmed that hybrid sporophytes could be obtained when the monoicous gametophyte was crossed with another male gametophyte, which indicates the eggs discharged by the monoicous gametophyte can really be fertilized by sperm, and thus they have the same sexual function as those discharged by the female gametophytes (Shan et al., 2021). They can also self-breed to give rise to doubled haploid (DH) sporophytes, albeit the fertilization rate of the eggs during selfing is much lower than during outcrossing. Despite the low fertilization rate in selfing, most of the derived sporophytes are morphologically normal, suggesting they are generated through self-fertilization (sexual reproduction) rather than parthenogenesis which usually results in a high ratio of abnormal parthenosporophytes (Shan et al., 2021).

A thorough interpretation of the life history is critical for conducting the cultivar breeding program. The versatile characteristics of *U. pinnatifida* life history provide not only challenges but also advantages for its fundamental and applied studies relevant to cultivar breeding.

## Establishment of Mapping Populations and Construction of Genetic Linkage Maps

In land crops such as rice, wheat and maize, the haploid phase of their life history is not free-living, and thus recombinant inbreeding lines (RILs) or DH lines need to be established to obtain true-breeding lines. Comparatively, the haploid gametophyte of kelp is free-living and thereby can be readily cultured and preserved *in vitro* under controlled conditions (Pang and Wu, 1996). This advantage provides a means to establish permanent mapping populations in kelp.

Meiosis-derived segregating haploid gametophyte families have been established in *U. pinnatifida*. Each gametophyte clonal line within a family is originated from mitotic propagation of a single meiospore and can be preserved for a long time. This kind of permanent mapping population has been used to construct an SNP-based high-density genetic linkage map using specific length amplified fragment (SLAF) sequencing in *U. pinnatifida* (Shan et al., 2015). Because the sex phenotype is expressed in the haploid phase, the population can be used to map sex-linked loci. Five SLAF and one microsatellite markers were found to be tightly linked to the sex phenotype (Shan et al., 2015).

The haploid gametophyte clonal lines have also been used to establish permanent sporophyte populations. A method of establishing an “immortalized F_2_” (IF_2_) population has been proposed in *U. pinnatifida*, in which the F_1_ segregating haploid gametophyte clonal lines, instead of the RILs or DH lines employed in land crops, are used for pairwise cross to obtain IF_2_ populations (Shan et al., 2020b). The method is also applicable to other kelp species. According to a cross experiment, it has also been found that the monoicous and parthenogenetic phenotypes of the parental gametophytes of *U. pinnatifida* are able to be inherited to the next gametophyte generation after meiosis within the hybrid sporophyte (Shan et al., 2021). Such an inheritable feature makes it possible to establish DH lines by exploiting phenotypes of monoicy and parthenogenesis. The availability of the methods of establishing IF_2_ and DH populations provide robust tools for analyses of quantitative trait loci (QTLs).

## DNA Markers Development and Their Application in Studies of Genetypic Variation and Genetic Structure

Markers such as random amplified polymorphic DNA (RAPD) and amplified fragment length polymorphism (AFLP) were adopted for genetic diversity analyses and germplasm identification in *U. pinnatifida* in earlier time (Wang et al., 2006; Li et al., 2013), but their use has been dramatically decreasing due to their drawback of dominant nature. Microsatellites and SNPs are currently the markers of choice for analyses of genetic diversity and structure. Totally 50 microsatellite markers (loci) have been developed and characterized for *U. pinnatifida* (Table 1) (Daguin et al., 2005; Shan et al., 2018). SNP markers have also been developed by double-digest restriction site-association DNA (ddRAD) sequencing or re-sequencing individuals from different populations (Guzinski et al., 2018; Graf et al., 2021). Both the microsatellite and SNP markers revealed that the genetic diversity and heterozygosity are higher in native natural and cultivated populations, but lower in introduced populations, showing evidence of founder effect in the latter. The unexpected high genetic diversity of the cultivated population is likely attributed to the mixture of hundreds of parental individuals of different origins in the seeding process (Shan et al., 2018; Li et al., 2020; Graf et al., 2021). Specific sequences from the mitochondrion (such as coding region of *cox3* and intergenic spacers between the *atp8* and *trnS* genes, between the *trnW* and *trnI* genes, and between the *tatC* and *tLeu* genes) and nucleus (e.g., internal transcribed spacer (ITS) of nuclear ribosomal DNA) (Voisin et al., 2005; Uwai et al., 2006a; Uwai et al., 2006b) have also been used to elucidate the genetic diversity in native and introduced populations globally. Combination analyses of sequences of maternal and bi-parental inheritance can provide complementary information on genetic relationship among populations, thereby being useful for tracing the origin of the introduced populations.

**TABLE 1:** Main genetic, transcriptomic and genomic resources available for *Undaria pinnatifida*.

Resource type	Brief introduction	References
Microsatellites	Twenty microsatellites isolated from an enriched library for tandem repeats, and 30 trinucleotide microsatellites developed through Illumina sequencing	Daguin et al. (2005), Shan et al. (2018)
Mitochondrial DNA sequence	Coding region of *cox3* and intergenic spacers between the *atp8* and *trnS* genes, between the *trnW* and *trnI* genes, and between the *tatC* and *tLeu* genes	Voisin et al. (2005), Uwai et al. (2006a), Uwai et al. (2006b)
Nuclear DNA sequence	Internal transcribed spacer (ITS) of nuclear ribosomal DNA	Uwai et al. (2006b), Endo et al. (2009)
SNPs	More than 10 thousand SNPs genotyped by using a dd-RAD sequencing method and applied in population genomics study; Millions of SNPs identified by re-sequencing the native and introduced populations	Guzinski et al. (2018), Graf et al. (2021)
Transcriptome of gametophytes	De novo transcriptome assembly from male and female gametophytes at vegetative and gametogenesis phases by using Illumina sequencing	Shan et al. (2015a)
Full-length transcriptome	A full-length transcriptome covering male and female gametophytes at vegetative and gametogenesis phases, and different tissues of the sporophytes obtained by using the PacBio sequencing platform	Shan et al. (2020a)
Genetic linkage map	A genetic linkage map constructed based on a segregating gametophyte family and SLAF sequencing, with five SLAF markers tightly linked to sex phenotype identified	Shan et al. (2015b)
Organelle genome	Plastid and mitochondrial genomes	Li et al. (2015), Zhang et al. (2015), (2016)
Nuclear genome	One male gametophyte from China and one sporophyte from Korea independently sequenced and characterized	Shan et al. (2020c), Graf et al. (2021)

Microsatellites and SNPs reveal prominent genetic structure in native and introduced populations of *U. pinnatifida*, even at the regional scale of the introduced area (e.g., Brittany, France), and SNPs have proved to be more robust in revealing the fine-scale genetic structure (Guzinski et al., 2018). Microsatellites have been used to evaluate the genetic connectivity between cultivated populations on a typical farm and the adjacent subtidal natural population of *U. pinnatifida*, and the result suggests scarce gene flow between them (Li et al., 2020). Such an evaluation is necessary in cultivation practice of seaweeds in order to avoid the reciprocal genetic pollution between farmed and natural populations.

## Transcriptomics and Genomics

Transcriptomic study is essential for gene discovery and functional annotation of the genome. De novo transcriptome assembly was conducted through Illumina-based RNA-seq of the male and female gametophytes at vegetative and gametogenesis phases (Shan et al., 2015). Putative genes, which encode the key enzymes involved in biosynthesis of important metabolites such as fucoidan, alginate, mannitol and laminarin, were identified. Subsequently, a full-length transcriptome was obtained from a collective sample consisting of both the sporophyte and the gametophyte phases using the single molecular real-time sequencing (Shan et al., 2020a). Illumina sequencing was then performed to identify the differentially expressed genes during the development of the sporophyll. Putative genes relevant to flagellar components, fucoidan biosynthesis and meiotic nuclear division were found to be significantly upregulated with development of the sporophyll.

Two nuclear genomes of *U. pinnatifida* have been obtained independently from a Chinese male gametophyte and a Korean sporophyte, both of which were from the cultivated population (Shan et al., 2020c; Graf et al., 2021). The genome from China is 511 Mb, and 502.8 Mb of the sequences are located on 30 pseudo-chromosomes with the assistant assembly of a Hi-C approach. The genome from Korea is larger with a total size of 634 Mb, and 462 Mb of them (72.7%) is anchored and ordered according to the linkage groups of the genetic map. The plastid and mitochondrial genomes of *U. pinnatifida* have also been sequenced (Li et al., 2015; Zhang et al., 2015; Zhang et al., 2016).

## Cultivar Breeding

Most of the authorized cultivars of *U. pinnatifida* have been bred using the selection method, including four Korean and two Chinese cultivars (Hwang et al., 2019). The breeding program usually consists of gametophyte clone cross and subsequent consecutive selection. Cultivars Haibao No.1 and No.2 were bred using such a selection strategy. The difference is that three hybrid lines resulting from gametophyte pair crosses were chosen and subjected to consecutive mixed hybridization and selection in Haibao No.1, and comparatively only one single hybrid line was chosen and followed by recurrent inbreeding and selection in Haibao No.2 (Xu et al., 2015; Shan et al., 2016).

Cross between gametophyte lines (line breeding) is another breeding strategy that has been developed based on the free-living characteristic of the kelp gametophyte. Heterosis can be fully exploited and the F_1_ hybrid sporophytes resulting from a single cross are homogeneous in genotype and phenotype (Pang, 1997; Pang et al., 1997; Shan and Pang, 2009). Hence, the F_1_ hybrid can be directly used as a commercial cultivar, greatly reducing the breeding period in comparison with the selection method. Line breeding conducted in *Undaria* includes interspecific hybridization between *U. pinnatifida* and *U. peterseniana*, and intraspecific cross between geographically separated individuals, between wild and cultivated individuals, or between cultivated individuals with contrasting traits (Hwang et al., 2012; Hwang et al., 2019; Niwa and Kobiyama, 2019). Triploid (3n) or tetraploid (4n) intraspecific hybrids have also been obtained by crossing between the diploid (2n) and haploid (n) gametophytes or between two diploid gametophytes. The triploid sporophytes were found to be sterile and superior in growth compared to diploid sporophytes, and tetraploid sporophytes were characterized with low-fertility and inferior growth (Zhang et al., 2007).

## Prospects on Cultivar Breeding

Collection and preservation of gametophyte stock resources are the first important work that needs to be done. The stock resources should encompass both farmed and wild populations. In previous breeding program particularly in China, wild resources have almost been disregarded due to its undesired agronomical traits (e.g., wrinkled fronds often observed in wild individuals) (Shan et al., 2018). In fact, there exist wild individuals with desirable traits that can be selected. Moreover, the local wild populations may comprise gene pools that are adapted to the local environment, and therefore are likely to be useful parental materials for breeding of cultivars suitable for cultivation in local waters (Li et al., 2020). In consideration of sustainable cultivation, the populations from warmer locations will be more important as they likely show better tolerance of higher temperature (Gao et al., 2012; Hu et al., 2021), which will confer better resilience of the cultivars to global ocean warming. For example, stock resources ought to be collected from southern distribution limit in East Asia such as Kagoshima of Japan, and Yushan and Zhoushan archipelagos of China (Tseng, 2001; Watanabe et al., 2014).

**FIGURE 1:**
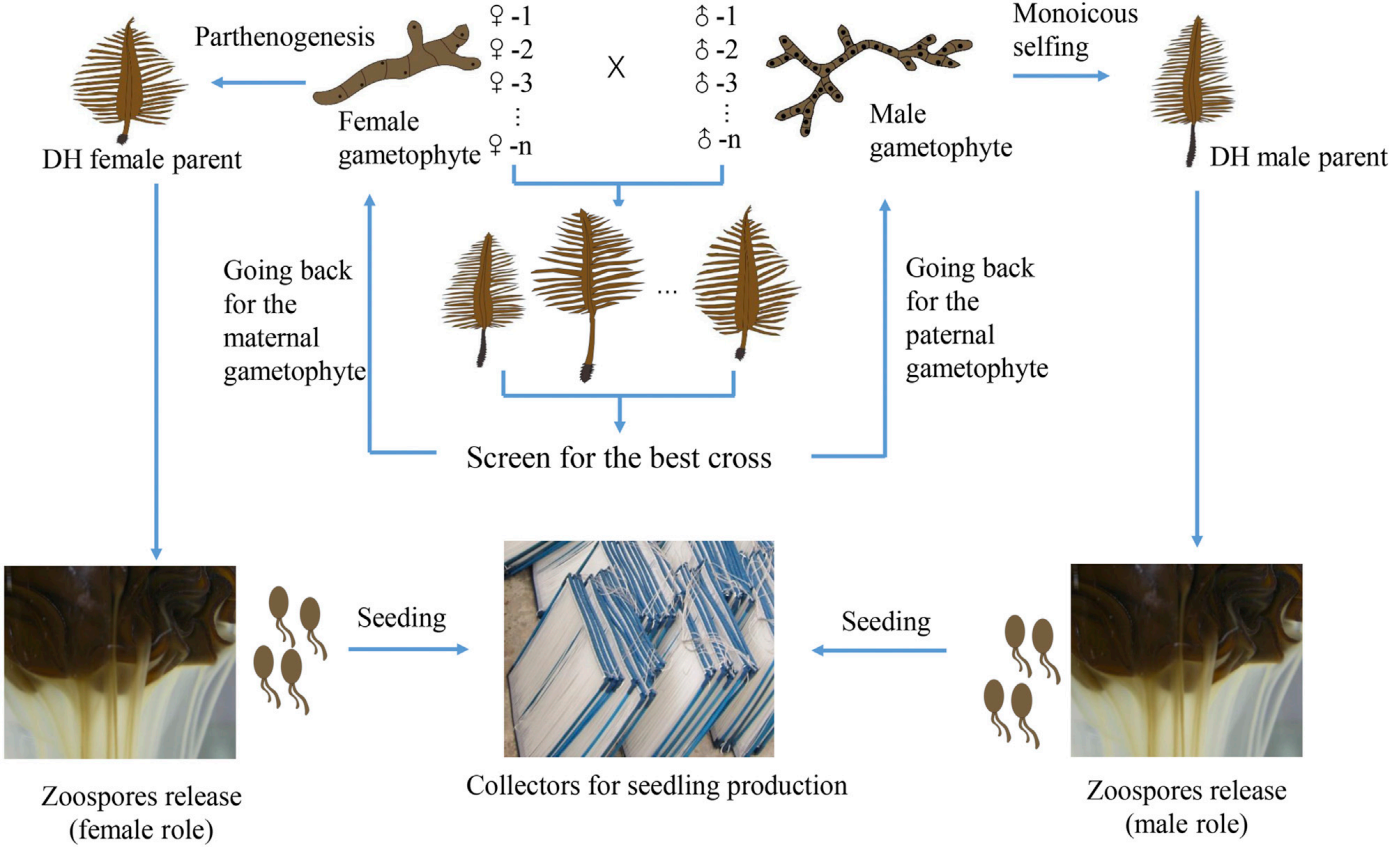
Schematic representation of line breeding (pair cross between gametophyte lines) and scaling up its application by exploiting monoicous and parthenogenetic phenotypes of certain parental gametophytes. After we screen for the best cross (with desirable traits), we go back for the corresponding paternal and maternal gametophyte lines. If either of them has the monoicous or parthenogenetic phenotype, DH sporophytes can be obtained. Hence, large quantities of zoospores can be utilized for seeding, followed by large-scale seedling production.

Traditional cultivar breeding methods are expected to play a central role in cultivar breeding in the near future. The recurrent mixed hybridization or inbreeding of superior parental sporophytes and targeted selection is a breeding strategy of low-cost that can exploit the additive gene effects (Goecke et al., 2020). The simple technical requirement makes it easy to be mastered by the producers. Line breeding method is time-efficient, resulting in the commercial hybrid cultivar with homogeneous traits. It also helps the breeders to protect their intellectual property of the cultivar because the growers have to come back to the breeders every year for the original superior F_1_ hybrid seedlings. What the breeders need to do is to keep the stock culture of the parental gametophyte clonal lines and propagate a large quantity of gametophyte filaments for seedling production. The challenge is to produce enough gametophyte biomass satisfied for large-scale cultivation. It may be partly tackled by using the DH sporophytes resulting from monoicous selfing and parthenogenesis of certain paternal and maternal gametophyte lines (Figure 1). Monoicy phenotype has been shown to be more common than expected in cultivated strains (Li et al., 2014; Shan et al., 2021). The fecundity of mature DH sporophytes is high, being able to release millions of spores with the same genotype as that of the corresponding parental gametophyte (Shan et al., 2013; Shan et al., 2021). Hence, DH sporophytes can be utilized in crossbreeding to scale up the production by circumventing the laborious and time-consuming propagation of gametophyte filaments. Another advantage of this method is that the zoospores released from the DH sporophytes can be easily seeded on the strings of the collectors, largely solving the detachment problem when gametophyte filaments are directly used for seeding (Li et al., 2017; Shan et al., 2021).

Genetic linkage and QTL mapping analyses can be conducted using the permanent mapping populations including DH and IF_2_. As quantitative traits of these populations can be repeatedly measured across different years and locations, it is possible to evaluate the environmental effects and the interaction between genotype and environment, thus making QTL analyses more accurate (Shan et al., 2020b; Shan et al., 2021). Genome-wide association analyses of morphological traits have recently been conducted in *Saccharina latissima* and one SNP was found to be associated with the stipe length (Mao et al., 2020). With the availability of the genome of *U. pinnatifida*, it is also feasible to conduct association mapping in this alga. Linkage and association mapping methods are complementary, and the appropriate approach may be chosen according to the purpose of the study. Identification of QTLs, particularly those of major effects, can accelerate the marker-assisted selection (MAS), which is performed depending on selection of individuals with specific DNA markers linked to or associated with the QTLs underpinning the desired trait. The more advanced breeding strategy such as genomic selection and epigenetics-mediated breeding may also be considered and developed to provide more alternatives for cultivar breeding (Goecke et al., 2020; Dalakouras and Vlachostergios, 2021).

A reverse genetics methodology which is based on the CRISPR-Cas9 gene-editing system has recently been established in the model brown alga *Ecotocarpus* sp. (Badis et al., 2021). Either biolistics or microinjection was shown to be efficient in delivering the introduction of CRISPR-Cas9 ribonucleoproteins into *Ectocarpus* cells, and mutations at specific target sites were generated. This method provides a robust tool for studying function of the genes and relating gene function to specific traits. If transferred to *U. pinnatifida*, the CRISPR-Cas9-based gene-editing method may be utilized to introduce the traits of interests by editing the relevant genes in this alga.
